# Molecular pathology of lymphoma and its treatment strategies: from mechanistic elucidation to precision medicine

**DOI:** 10.3389/fimmu.2025.1620895

**Published:** 2025-07-09

**Authors:** Zhongyu Wang, Shuai Feng, Xiangmei Yao, Renbin Zhao, Yujin Li, Maofeng Zheng, Zengzheng Li, Yajie Wang

**Affiliations:** ^1^ Department of Hematology, The First People’s Hospital of Yunnan Province, Affiliated Hospital of Kunming University of Science and Technology, Kunming, China; ^2^ Yunnan Province Clinical Research Center for Hematologic Disease, The First People’s Hospital of Yunnan Province, Kunming, China; ^3^ Yunnan Provincial Clinical Medical Center for Blood Diseases and Thrombosis Prevention and Treatment, The First People’s Hospital of Yunnan Province, Kunming, China; ^4^ Yunnan Atherosclerosis Cooperation Base of Chinese and Western Medicine, The First People’s Hospital of Yunnan Province, Kunming, China; ^5^ School of Medicine, Kunming University of Science and Technology, Kunming, China; ^6^ Xiyuan Hospital, China Academy of Chinese Medical Sciences, Beijing, China; ^7^ Graduate School, Beijing University of Chinese Medicine, Beijing, China

**Keywords:** lymphoma, precision medicine, immunotherapy, nanotechnology, artificial intelligence

## Abstract

Lymphoma is a highly heterogeneous hematologic malignancy characterized by intricate molecular and pathological mechanisms. Key mechanisms contributing to its complexity include malignant clonal evolution driven by somatic mutations, epigenetic modifications affecting gene regulation and cellular behavior, and dynamic tumor microenvironment remodeling. These factors collectively undermine the efficacy of conventional therapeutic strategies. Differences in the molecular mechanisms of different subtypes lead to heterogeneity in treatment response and recurrence of drug resistance. Current and future investigative priorities emphasize molecular stratification, precision diagnosis and therapeutic strategies, advancement of novel diagnostic tools, and the implementation of artificial intelligence (AI) for integrative analysis of high-dimensional biological data. Moreover, emerging areas such as microbiome-targeted interventions are being explored to improve clinical outcomes and support the evolution of precision oncology in lymphoma treatment.

## 1 Introduction

Lymphoma is one of the most commonly diagnosed malignant neoplasms globally, with an estimated 89,000 new cases and over 21,000 related deaths reported in 2024. Pathologically, they are classified into Hodgkin’s lymphoma (HL) and non-Hodgkin lymphoma (NHL). NHL mainly comprises B-cell lymphomas such as diffuse large B-cell lymphoma (DLBCL), follicular lymphoma (FL), metachronous lymphoma (MCL), and T-cell lymphomas such as peripheral T-cell lymphoma (PTCL), interstitial large-cell lymphoma (ALCL), and angio-immunoblastoid T-cell lymphomas (AITL), and NK-cell lymphomas (1). HL is relatively uncommon and has considerable regional variability in incidence, with the classical subtype representing the most frequently observed form (2). The molecular pathogenesis of lymphoma subtypes is highly heterogeneous. For example, cell-of-origin (COO)-based classification of DLBCL distinguishes between germinal center B-cell-like (GCB) and non-germinal center B-cell-like (non-GCB) subtypes, each characterized by distinct gene expression profiles, including differential expression of markers such as *BCL6* and *MYC* (3). Double-hit lymphoma (DHL), defined by rearrangements involving *MYC* and *BCL2* and/or *BCL6*, displays a highly aggressive clinical phenotype, elevated proliferative potential, resistance to conventional therapeutic approaches, and very poor prognosis (4, 5). In-depth analysis of molecular features can provide a key basis for developing individualized treatment strategies. Currently, the treatment of lymphoma faces two major challenges: drug resistance and relapse. Tumor cells may be resistant to drugs through multiple escape mechanisms, and invasiveness and drug resistance may increase after relapse.

This paper aims to provide a comprehensive and systematic analysis of the molecular mechanisms underlying lymphoma and to examine current therapeutic strategies with particular emphasis on the contributions of molecular pathology to diagnosis, prognostic evaluation, and therapeutic decision-making. The study further addresses limitations in existing treatment modalities. It highlights future research priorities, including developing next-generation targeted therapies, optimization of immunotherapy protocols, investigating combinatorial treatment strategies, and incorporating nanotechnology and artificial intelligence (AI). The overarching goal is to advance therapeutic efficacy and improve patient survival while establishing a theoretical basis for the continued development of precision medicine in lymphoma care.

## 2 Molecular pathological mechanisms of lymphoma

### 2.1 Genomic diversity and key driving factors

#### 2.1.1 Genes with high-frequency mutations: MYC, BCL2, TP53, NOTCH1

Recent advancements in molecular pathology research have identified recurrent genetic alterations associated with lymphoma. Frequent mutations such as *MYC* gene rearrangements, *BCL2* translocations, inactivating mutations in *TP53*, and truncating mutations in the PEST domain of *NOTCH1* have been increasingly recognized. These mutations play a key role in the occurrence and development of lymphoma. *MYC* gene rearrangement disrupts the metabolic homeostasis of oxidative phosphorylation, leading to uncontrolled tumor proliferation. Such cases are often accompanied by genomic instability and reduced chemosensitivity (6). FL is characterized by an inherited alteration of IGH-BCL2 translocation, which puts the *BCL2* gene under the control of immunoglobulin enhancers, resulting in overexpression. *BCL2*-positive patients typically respond poorly to chemotherapy and have shorter survival (7). Mutations or inactivation of the *TP53* gene are relatively common in relapsed and refractory lymphoma. Such gene abnormalities are closely associated with increased tumor aggressiveness, treatment tolerance, and a high risk of recurrence, which seriously affect the prognosis of patients (8). NOTCH1 is a driver of MCL (9). Target gene networks such as HES1/HEY1 regulate cell proliferation and differentiation, angiogenesis, and drug resistance development, becoming key drivers of aggressive disease progression.

#### 2.1.2 Structural variations: chromosomal translocations and copy number variations

Genomic structural abnormalities can contribute to the malignant transformation of lymphoma. A prominent example is chromosomal translocation, with the *IGH-BCL2* translocation being the most frequently observed in follicular lymphoma. This translocation leads to *BCL2* gene overexpression under the control of the IGH enhancer, therefore inhibiting apoptosis and facilitating lymphoma initiation and progression. Copy number variations (CNVs) are also quite common. For instance, the deletion of the cyclin-dependent kinase inhibitor 2A (*CDKN2A*) gene is an independent factor associated with poor progression-free survival (PFS) and overall survival (OS) (10). Such patients often have a poor prognosis and reduced responsiveness to chemotherapy (11). Deletion of the p16INK4a protein encoded by *CDKN2A* leads to an uncontrolled cell cycle, which promotes tumor cell proliferation (12). These structural variants not only reveal the core mechanism of lymphoma progression but also provide molecular loopholes for targeted therapy.

### 2.2 Epigenetic regulatory network

Epigenetic regulatory networks show intricate interactions with non-coding RNAs *via* DNA methylation and covalent histone modifications, thus establishing multilayered modulation of gene transcription. Among these modifications, DNA methylation and histone methylation are the most prevalent. They are frequently associated with the transcriptional silencing of tumor suppressor genes and the inappropriate activation of proto-oncogenes, contributing to lymphomagenesis and disease progression. Dysregulation or mutations in genes related to DNA methylation (such as *DNMTs, TET2, IDH2*) and genes associated with histone methylation (such as *EZH2, KMT2D*) have been observed, and most of these alterations are associated with poor prognosis (13). DNA methyltransferases (DNMTs) are key factors in regulating DNA methylation. Among the DNMT family, *DNMT1*, *DNMT3A*, and *DNMT3B* are associated with tumourigenesis (14). Deficiency of *DNMT1* leads to abnormal self-renewal, niche preservation, and cell differentiation of hematopoietic stem cells (HSCs), especially to the myeloid lineage. Moreover, deficiency of *DNMT3A* and *DNMT3B* impairs the self-renewal ability of HSCs (15). Although *DNMT3A* is less frequently overexpressed in lymphoma than *DNMT1* and *DNMT3B*, it shows the highest mutation frequency among the DNMT family. Its deletion has increased mast cell reactivity and exacerbated inflammatory responses *in vivo* (16, 17). Poole CJ et al. reported that D*-MYC* induces the overexpression of *DNMT1* and *DNMT3B*, which promotes tumor maintenance (18).

Post-translational modifications of histones, such as acetylation and methylation, play a key role in regulating the three-dimensional organization of chromatin. These reversible covalent changes contribute to the structural flexibility and dynamic remodeling of higher-order chromatin architecture. Considering histone acetylation as an example, as the core catalytic component of the Polycomb Repressive Complex 2 (PRC2) complex, EZH2 protein mediates epigenetic regulation by catalyzing the trimethylation of lysine 27 on histone H3 (H3K27me3). This modification can directly inhibit the transcriptional activity of target genes and collaborate with DNA methyltransferases to participate in gene silencing (19, 20). Importantly, gain-of-function mutations at the *Y641* locus are detected in about 40% of B-cell lymphoma patients. These mutations significantly enhance abnormal silencing of oncogene-related genes by altering enzymatic kinetics, representing one of the key molecular mechanisms driving tumorigenesis (21).

Long noncoding RNAs like Plasmacytoma Variant Translocation 1 (*PVT1*) play a key role in epigenetic regulation. Traversa D et al. found that this RNA molecule is often overexpressed in lymphomas. It activates the *MYC* gene *via* chromosomal translocations and relieves *MYC* inhibition by sponging miRNAs, thus driving tumor proliferation and survival (22). Moreover, *PVT1* has linear and circular transcriptional variants. Simultaneous silencing of linear *PVT1* and its circular isoform circPVT1 suppresses Burkitt lymphoma progression, whereas exogenous circPVT1 overexpression offsets the loss of endogenous circular transcripts and enhances malignant behavior in B-cell lymphomas (23). ceRNA competitively binds to miRNAs with mRNAs by sharing miRNA response elements (MREs), forming a “ceRNA-miRNA-mRNA” regulatory network. This network can upregulate *MYC* and *BCL2,* restore *PTEN* expression, and intervene in the PI3K/AKT pathway, affecting tumor cell proliferation and chemoresistance (24). *PVT1* is located near *c-MYC*. Its circular product, circPVT1, co-localizes with *c-MYC* and has a stable circular structure. It can interact with RNA-binding proteins to regulate cell processes and promote lymphoma development (25). Since *c-MYC* is highly activated in Burkitt lymphoma, circPVT1 may rely on *c-MYC* to drive proliferation. Meanwhile, as a miRNA sponge, circPVT1 may competitively target miR-15/16 and other miRNAs that target *BCL2* through the ceRNA mechanism, relieving the inhibition of *BCL2/c-MYC* (26).

### 2.3 Tumor microenvironment interactions

**Figure 1:**
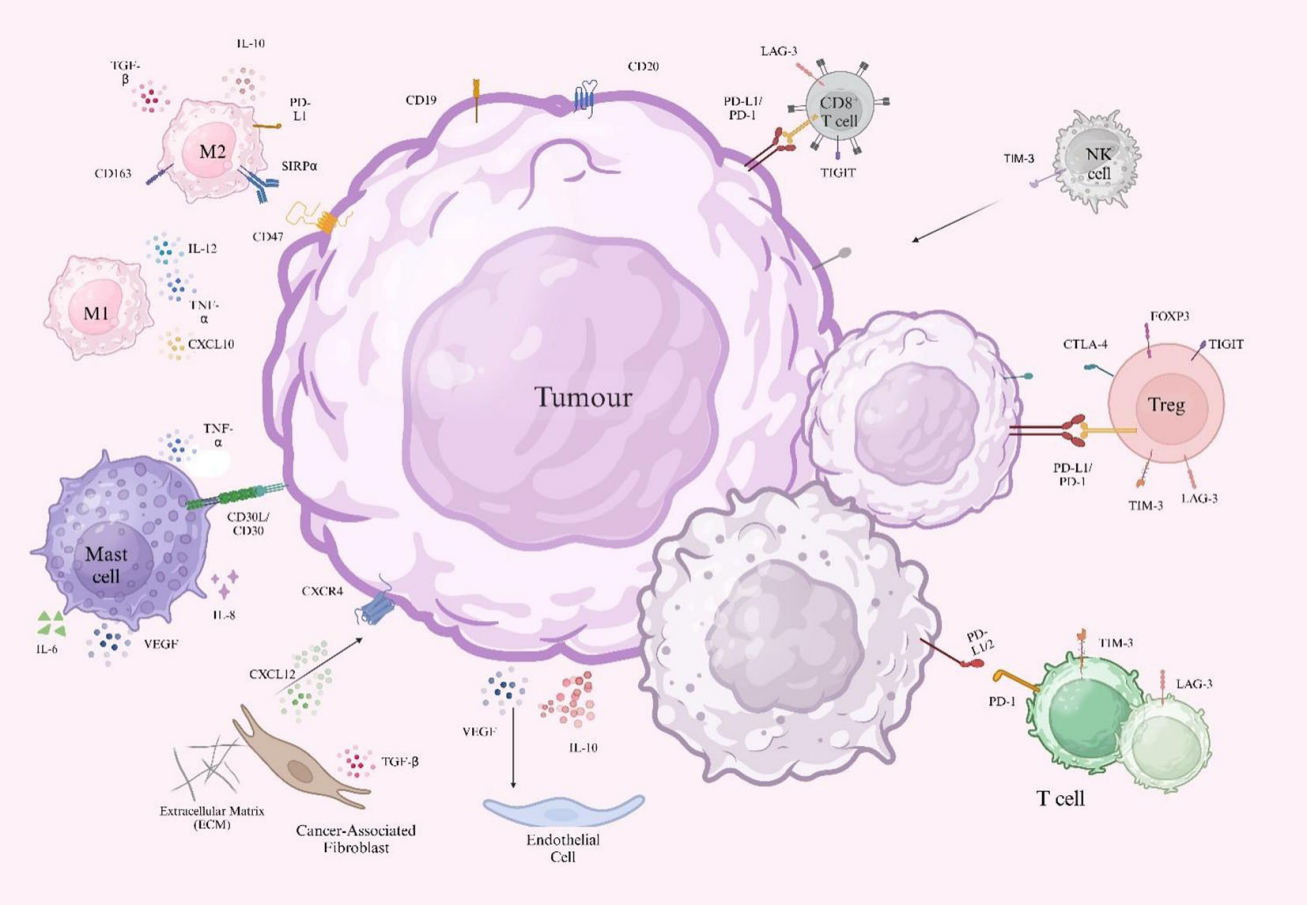
Schematic representation of cellular components in the tumor microenvironment. Tumor cells express surface markers (CD19 and CD20) and secrete immunosuppressive factors (IL-10 and VEGF). Tumor-derived PD-L1 binds to PD-1 on CD8^+^ T cells, inhibiting T cells. CD47 interacts with SIRPα on macrophages, suppressing phagocytosis and supporting tumor survival. Tregs express FOXP3, while M2 macrophages are marked by CD163^+^ and contribute to immunosuppression. CAFs release CXCL12, which binds CXCR4 on tumor cells to promote migration and homing and also secrete TGF-β, which modulates tumor growth and therapeutic resistance. CD31 marks endothelial cells and contributes to aberrant angiogenesis. ECM releases fibronectin and collagen, promoting fibrosis. M1 macrophages secrete IL-12 and TNF-α to increase T cell-mediated immunity, whereas M2 macrophages promote tumor growth *via* IL-10 and TGF-β-induced T cell exhaustion. Mast cells produce IL-6, IL-8, TNF-α, and VEGF, facilitating tumor proliferation, angiogenesis, and immune suppression.

The tumor microenvironment (TME) (**Figure 1**) is a highly dynamic and intricate biological environment composed of malignant cells and various stromal elements, with key components including immune cells (e.g., T cells, macrophages, regulatory T cells), vascular endothelial cells, and the extracellular matrix (ECM) (27). Tumor-associated macrophages (TAMs) show significant functional heterogeneity in the lymphoma microenvironment. M1-type macrophages activate T-cell immune responses by secreting pro-inflammatory cytokines like IL-12 and TNF-α, while M2-type macrophages induce T-cell exhaustion by releasing inhibitory factors such as IL-10 and TGF-β. The dynamic balance between these two directly affects tumor progression and treatment response (28). Specific overexpression of enolase 2 (ENO2) promotes glycolytic metabolic reprogramming *via* activating the GSK3β/β-catenin/c-Myc signaling pathway, thus inducing macrophage polarization toward the M2 phenotype and creating a tumor-promoting microenvironment (29). When PD-L1 inhibitors are used in combination with lenalidomide, they not only convert PD-1^+^ M2 TAMs to an M1 phenotype with phagocytosis through immunometabolic reprogramming but also block the IL-10-PD-1/PD-L1 immunosuppressive axis and restore the proliferation of CD4^+^/CD8^+^ T cells (30). The CTLA-4/CD86 costimulatory signaling axis also represents a PD-1-independent immune escape pathway, particularly prominent in cHL. Residual CTLA-4^+^ T cells and CD86^+^ TAMs persist after PD-1 blockade, suggesting that combining CTLA-4 inhibitors may emerge as a new strategy to overcome drug resistance (31).

Tumor cells evade immune surveillance *via* diverse mechanisms. Antigen escape arises when tumor cells undergo mutation, downregulation, or loss of target antigens, impairing CAR-T cell recognition (32, 33). TME contributes to epigenetically driven T-cell exhaustion, diminishing normal T-cell activity and limiting durable responses to PD-1 inhibitor therapy (32, 33). Furthermore, tumors establish a comprehensive immunosuppressive network by recruiting regulatory T cells (Tregs), myeloid-derived suppressor cells (MDSCs), and secreting immunoinhibitory cytokines such as TGF-β and IL-10, which suppress T-cell function at multiple levels (32, 33). These processes contribute to therapeutic resistance and disease relapse.

Metabolic competition plays a key role in reshaping the TME. Tumor cells undergo glycolysis by massively uptaking glucose *via* the Warburg effect, leading to lactic acid accumulation and increased acidity in the microenvironment. This not only directly inhibits T-cell function but also promotes the expansion of Tregs and regulatory B cells (Bregs) by activating immunosuppressive signaling pathways (such as TGF-β and IL-10) (34). Mishina T et al. reported that elevated expression levels of TGF-β and IL-10 in patients with DLBCL were significantly correlated with R-CHOP treatment failure. These findings indicate that targeting metabolic–immune interactions may represent a pivotal strategy for improving therapeutic outcomes (35).

### 2.4 Clonal evolution and drug resistance mechanisms

Adaptive resistance in tumors, driven by clonal evolution, is a significant challenge in cancer therapy. This phenomenon primarily arises from the interaction between genomic alterations and epigenetic reprogramming. Liquid biopsy technology enables real-time monitoring of spatiotemporal changes in resistant clones by jointly analyzing circulating tumor DNA (ctDNA) methylomes and mutation profiles. It can accurately capture adaptive mutations like BTK-C481S. Furthermore, it allows quantitative analysis of phenotypic plasticity indices, revealing the process of drug-resistant phenotype transformation mediated by DNA methylation and histone modification reprogramming under chemotherapy pressure (36). Studies analyzed 73 cases of DLBCL and found that pre-treatment ctDNA levels were an independent prognostic factor. The dynamic clearance rate of ctDNA has shown comparable efficacy to PET-CT in assessing therapeutic response. The presence of residual ctDNA after treatment correlates with a high risk of disease recurrence. Moreover, ctDNA testing overcame the limitations of tumor spatial heterogeneity and enabled the detection of an additional 170 driver mutations not identified through conventional tissue biopsy (37).

In single-cell multi-omics research, Tran N et al. used single-cell chromatin accessibility analysis (scATAC-seq) to study epigenetic changes in tumor cells during chemotherapy. They found that subclones acquire stem-like properties through abnormal activation of the KDM6A demethylase (38, 39). During chemotherapy, lymphoma cells can acquire chemoresistance by upregulating drug resistance-related genes through epigenetic mechanisms such as DNA methylation and histone modification changes. The development of evolutionary prediction models offers a strategic approach for optimizing intervention timing and enabling precise therapeutic targeting before the expansion of resistant clonal populations.

## 3 Innovative treatment strategies

**Figure 2:**
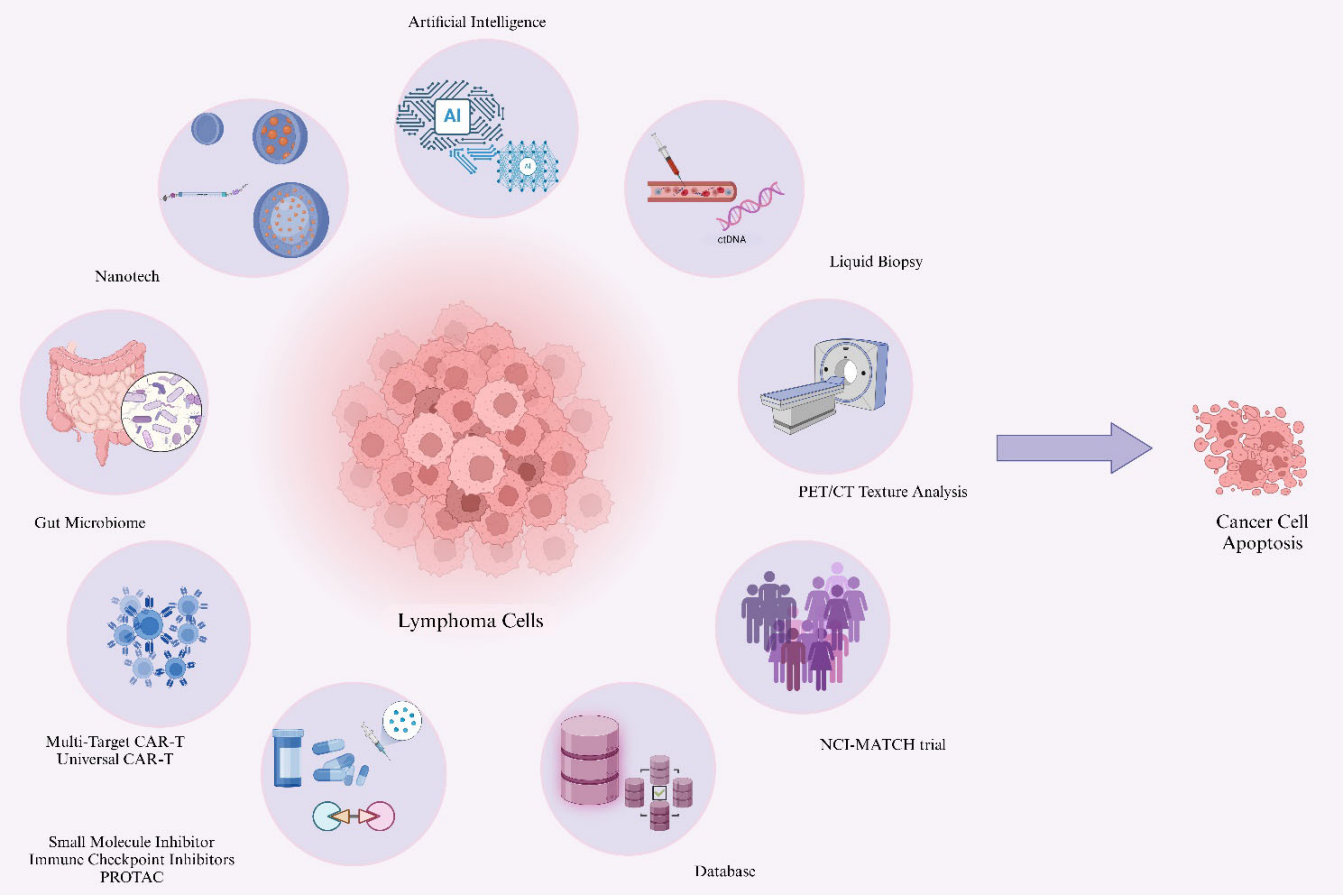
Innovative treatment strategies.

Lymphoma treatment strategies are experiencing a paradigm shift from conventional approaches toward precision medicine. Based on first-line chemoimmunotherapy, emerging therapeutic interventions for relapsed or refractory cases are being developed across several key dimensions (**Figure 2**).

### 3.1 Breakthroughs in targeted therapy

#### 3.1.1 Small molecule inhibitors: Bruton’s Tyrosine Kinase, BCL2, and Enhancer of Zeste Homolog 2 inhibitors

In cancer treatment, targeted molecular therapies are used not only as first-line treatments but also often as second-line regimens. Their mechanism of action involves the selective targeting of intrinsic molecular susceptibilities within malignant cells. These therapies include small-molecule inhibitors and protein degradation technologies. Common small-molecule inhibitors include BTK inhibitors, BCL2 inhibitors, and EZH2 inhibitors. The main action of BTK inhibitors has been systematically described by the teams of Broccoli A and Tian G (40, 41). Ibrutinib is an oral Bruton’s tyrosine kinase (BTK) inhibitor. It irreversibly inhibits the B-cell receptor (BCR) signaling pathway by covalently binding to the cysteine (Cys) 481 site, marking a milestone in treating B-cell tumors. First-generation drugs, such as Ibrutinib, have been approved by the European Medicines Agency (EMA) to treat relapsed or refractory mantle cell lymphoma (R/R MCL). Clinical evidence indicates that the timing of therapeutic intervention exerts a substantial influence on patient prognosis (42, 43). The SYMPATICO study showed that the combination of Venetoclax can extend the median PFS of relapsed MCL patients to 31.9 months (44). Patients treated with ibrutinib at the first recurrence had a 2.5-fold improvement in median PFS compared with late-line use (25.4 vs 10.3 months) (45). To address the therapeutic limitations of drug resistance, Chen Q et al. conducted a study demonstrating that the CDK1 inhibitor RO-3306 increases sensitivity to BTKi in DLBCL. These findings propose a novel therapeutic strategy for overcoming resistance mediated by the C481S mutation (46). For first-generation drugs like Ibrutinib, clinical response declines due to resistance mutations such as C481S. Second-generation inhibitors Acalabrutinib and Zanubrutinib address this by optimizing kinase selectivity to improve specificity and reduce off-target effects. Zanubrutinib improves oral bioavailability by 3.9% compared to Ibrutinib, showing higher ORR and longer PFS in relapsed MCL (47, 48). Chihara et al. reported that the combination of Ibrutinib with PD-1 inhibitors yielded an ORR of 77.8% in patients with primary or secondary central nervous system (CNS) lymphoma. However, treatment was associated with grade 3 to 4 hematologic toxicities (49). In the future, comprehensive molecular profiling of CNS lymphoma is needed to refine diagnostic criteria and optimize therapeutic strategies. Furthermore, dynamic monitoring of ctDNA holds the potential for guiding timely interventions against resistant subclones, thus enabling more precise therapeutic targeting. Venetoclax is a highly selective BCL-2 protein antagonist. It works by competitively binding to the BH3 domain of BCL-2. This relieves BCL-2’s inhibition of pro-apoptotic proteins like BAX/BAK. It also restarts the tumor cell apoptosis program (50). BCL-2 overexpression often coexists with EZH2 gain-of-function mutations. Targeted combination with Tazemetostat (an EZH2 inhibitor) can synergistically induce dual regulation of the epigenetic-apoptotic axis (51, 52).

Coughlin CA et al. revealed that *BCL10* mutations in DLBCL contribute to treatment resistance *via* two distinct mechanisms. First, they activate the NF-κB signaling pathway and establish a positive feedback loop involving IL-6 and STAT1/2, which increases the expression of anti-apoptotic BCL-2 family proteins such as BCL-xL and BFL1, thus conferring resistance to Venetoclax monotherapy. Simultaneously, these mutations alter the BCR signaling network, suppressing the inhibitory effects of BTK inhibitors on downstream kinases (53). To address this resistance mechanism, the combination of non-covalent BTK inhibitors Pirtobrutinib and Venetoclax reveals significant synergistic effects. It blocks NF-κB/AP-1 transcriptional activation mediated by *BCL10* mutations while suppressing the expression of anti-apoptotic proteins BCL-2 and MCL-1. This significantly inhibits the growth of *BCL10*-mutated lymphoma cells, showing potent antitumor activity and providing a new strategy to overcome *BCL10* mutation-related drug resistance (53).

The EZH2 inhibitor Tazemetostat targets the catalytic subunit of the histone methyltransferase PRC2 complex. It competitively inhibits its binding to the cofactor S-adenosylmethionine (SAM), thus blocking *H3K27me3*-mediated epigenetic silencing (54). In FL and DLBCL, *EZH2* gain-of-function mutations (i.e., *Y641F*) drive silencing of tumor suppressor genes like *CDKN2A* by improving H3K27me3 catalytic efficiency. These mutations are significantly associated with tumor progression and poor prognosis (55). Izutsu K et al. used Tazemetostat as monotherapy for *EZH2*-mutated FL, yielding 70.6% ORR (56). This demonstrated good long-term efficacy and safety, supporting its use as a third-line treatment option. The ongoing SYMPHONY-1 Phase III trial (NCT04224493) for relapsed/refractory FL evaluates its synergistic effects when combined with lenalidomide-rituximab. Importantly, the *EZH2* resistance mutation profile reveals dynamic evolutionary characteristics. A recent study identified that the *W113C* mutation within the SET domain increases the binding affinity of SAM, thus attenuating the inhibitory potency of Tazemetostat by altering the positioning and structural stability of residue R685 (55). This resistance mechanism shows molecular heterogeneity compared to the classic *Y641F* mutation. This finding highlights the need for systematic screening of non-hotspot *EZH2* mutations in clinical practice and drives the development of new-generation PRC2 inhibitors. Currently, MAK683, CPI-1205, SHR2554, PF-06821497, and other agents are in Phase 2 or 3 antitumor trials. Efficacy validation of Tazemetostat combination regimens in DLBCL (NCT05618366 and NCT05604417) and analysis of resistance mechanisms will provide critical evidence-based support for targeted therapy.

#### 3.1.2 Protein degradation technologies

Proteolysis-Targeting Chimera (PROTAC) technology constructs bifunctional molecules composed of a target protein-ligand and an E3 ligase ligand. These molecules form a ternary complex of target protein-PROTAC-E3 ligase, triggering ubiquitination of the target protein and proteasome-mediated specific degradation (57). This technology has demonstrated significant advantages in targeting oncoproteins (BCL6 and MYC) refractory to traditional therapies (58). Malarvannan M et al. comprehensively evaluated the design protocols and optimization strategies employed in developing PROTACs (59). As a representative example, the BCL6-specific degrader DZ-837 was rationally designed based on the N-phenyl-4-pyrimidineamine scaffold. It can effectively reduce BCL6 protein levels in DLBCL cells. It can also be used in combination with Ibrutinib to overcome resistance mutations. PROTAC molecules directed against *MYC* have shown dual therapeutic efficacy in mouse models: prolonging survival and inhibiting tumor growth (60). Compared with gene editing technologies like CRISPR-Cas9, the PROTAC system has unique, reversible regulatory properties. Its mechanism of action does not rely on permanent genomic modifications. It provides more clinical application potential in terms of operational cycle, cost control, and off-target risk (61). Fu et al. further expanded this platform and successfully constructed an *EZH2*-targeted molecular library. Among these agents, the bifunctional degrader ZJ-20 induces the degradation of the EZH2 enhancer, thus facilitating the disassembly of the entire PRC2. It shows excellent antiproliferative activity and favorable pharmacokinetic properties (62). Studies have developed new PROTAC molecules based on the reversible non-covalent BTK inhibitor ARQ531. These molecules can effectively circumvent resistance mechanisms mediated by *C481S* mutations. Their efficacy is significantly higher than traditional covalent BTK-PROTACs (63). Particularly, the innovative STAT3D PROTAC series molecules conjugate a STAT3-specific decoy with thalidomide. This not only efficiently reduces STAT3 protein levels but also precisely inhibits the expression of key oncogenes regulated by *STAT3*, such as *BCL2L1, CCND2*, and *MYC.* As a result, it suppresses lymphoma cell proliferation while inducing cell apoptosis (64). This targeted degradation strategy showed minimal off-target activity against other members of the STAT family, such as *STAT1* and *STAT5*, thus contributing to improved therapeutic specificity and reinforcing the direct anti-tumor efficacy in B-cell lymphoma. Together, these developments have validated the important application value of PROTAC technology in cancer therapy (64).

### 3.2 Frontiers in immunotherapy

#### 3.2.1 Era of cell therapy 2.0

Recently, CD20-directed therapy has been the core strategy in managing B-cell lymphoma. The following section provides a systematic overview of conventional therapeutic approaches targeting CD20, CD30, and CD52 antigens in treating lymphoma (**Table 1**).

**Table 1:** Advantages and disadvantages of conventional CD20/CD30/CD52-targeted therapy for lymphoma.

Target	Representative drugs	Indications	Advantages and disadvantages	Notes
CD20	Rituximab	DLBCL, FL, CLL, MCL	Advantages: Broad applicability; Disadvantages: High rate of drug resistance, risk of HBV reactivation.	(65)
	Ofatumumab	FL	Advantages: Effective for low CD20 expression. Disadvantages: High risk of infection.	(66, 67)
	Obinutuzumab	CLL, FL	Advantages: PFS compared to rituximab. Disadvantages: More frequent infusion reactions.	(68)
CD30	Brentuximab vedotin	cHL, ALCL	Advantages: High targeting specificity, significantly prolongs the survival period. Disadvantages: Neuropathy, myelosuppression.	(69)
CD52	Alemtuzumab	Cutaneous T-Cell Lymphoma (CTCL)	Advantages: Potently clears malignant lymphocytes. Disadvantages: Extremely high risk of infection.	(70)

Advancements in multi-targeted therapeutic strategies are driving significant innovation in the clinical management of lymphoma. In this context, Esquinas et al. engineered novel chimeric CD79b-targeted CAR-T cells. They found that these CAR-T cells can induce CD79b and CD19 co-downregulation in NHL cells. Among them, CARLY3 has become an advantageous candidate for NHL treatment. It has excellent targeting specificity, durable tumor clearance ability, and minimal antigen loss rate (71).

Traditional single-target CAR-T has an antigen escape defect. Dual-target CAR-T (CD19/CD22) addresses this by significantly reducing recurrence risk through a synergistic targeting mechanism. It shows improved efficacy in relapsed/refractory B-cell lymphoma (72). CD20×CD3 bispecific antibodies are novel T cell-engaging antibodies (such as glofitamab and epcoritamab). They are approved by the FDA/EMA for treating DLBCL and FL. However, approximately half of the patients exhibit primary resistance (73, 74). A retrospective analysis by Kyvsgaard ER et al. identified NOTCH1 mutation-induced loss of CD20 antigen expression as a critical mechanism contributing to therapeutic resistance, highlighting the role of genetic alterations in modulating treatment efficacy (74). The novel bispecific antibody TG-1801 targets both the CD47-SIRPα axis and CD19. This improves the ADCC/ADCP effects of anti-CD20 antibodies. It also achieves multi-dimensional antitumor activity by regulating the GPR183-PI3Kδ pathway (75). Kolbe C et al. proposed a combinatorial therapeutic strategy involving anti-CD20 bispecific antibodies with CD39/CD73 inhibition. This regimen improves therapeutic efficacy by counteracting adenosine-mediated immunosuppression, restoring immune effector function within the TME. It increases tumor killing rates by 2.1-3.5 fold, CD8^+^ T cell expansion by 3.8 fold, and IFN-γ secretion by 4.2 fold. Patients with high CD39-expressing effector memory T cells (TEM) show a 67% higher response rate. This suggests that CD39 may serve as a predictive biomarker for efficacy (76). Resistance to BTK inhibitors (BTKi) and CAR-T is closely related to *Myc* pathway activation, with HSP90AB1 and CDK9 as key regulatory nodes (77). Jiang V et al. reported that the CDK9 inhibitor enitociclib effectively inhibits protein synthesis and induces apoptosis, successfully overcoming resistance to sequential therapies in a MCL model (78). A combined inhibition approach targeting both HSP90 and CDK9 offers a promising strategy for addressing therapeutic resistance. Moreover, CD19/CD70 dual-target CAR-T therapy achieved complete remission in patients with relapsed/refractory primary central nervous system DLBCL, sustaining 17 months of disease-free survival without neurotoxicity, highlighting the potential of multi-targeted immunotherapies (79). These advances, comprising targeted therapies, immune modulation, and resistance reversal, have significantly enhanced the precision treatment landscape for lymphoma and laid a solid foundation for future clinical translation.

Clinical trials for novel monoclonal antibodies, bispecific antibodies, and CAR-T therapies are currently being conducted globally. The following table (**Table 2**) summarizes some representative ongoing trials (March 2025).

**Table 2:** All data are sourced from ClinicalTrials.gov.

Trial ID	Intervention	Phase	Target population	Primary endpoint	Status	Estimated completion time	Location
NCT06824701	Tazemetostat +Zanubrutinib + anti-CD20 monoclonal antibody	1b	r/r B-NHL	Maximum Tolerated Dose (MTD)	Recruiting (Not yet recruiting)	2032-01	United States
NCT06565689	YK012 (Targeting CD19×CD3)	1	r/r B-NHL	Adverse Event (AE), Dose-Limiting Toxicity (DLT), MTD	Recruiting	2026-06-30	Beijing, China
NCT06563596	Epcoritamab,+Zanubrutinib+ Rituximab	II	R/R FL	Complete Metabolic Response (CMR) Rate	Recruiting	2027-03-01	United States
NCT06532643	Anti-CD20/CD30 CAR-T		R/R Lymphoma	Safety and Tolerability, Manufacturing Feasibility	Recruiting	2025-09-01	Anhui, China
NCT06464185	CD3-CD20 Bispecific Antibody in Combination with CD19 CAR-T		B-NHL	Incidence and Severity of AEs	Recruiting	2026-04-30	Tianjin, China
NCT06284122	Mosunetuzumab+ Lenalidomide+ Rituximab	III	FL	PFS	Recruiting	2028-11	Belgium, France, Germany, Portugal, Spain
NCT06026319	CD79b ×CD19 CAR T	I	r/r NHL	Incidence of AEs and Incidence of DLTs	Recruiting	2027-01-01	Massachusetts, United States
NCT06014762	P-CD19CD20-ALLO1 (Targeting CD19 CD20)	I	r/r B-Cell Malignancies	DLT	Recruiting	2029-03	United States
NCT05990465	LV20.19 CAR-T + Pirtobrutinib	I	r/r B-Cell Malignancies	Number of AEs	Recruiting	2026-07	Wisconsin, United States
NCT05607420	UCART20x22		r/r B-NHL	Dose Exploration and DLT	Recruiting	2027-11	United States, France, Spain
NCT05421663	JNJ-90014496 (Targeting CD19 × CD20)	Ib	B-NHL	AE	Recruiting	2026-05-29	United States, Canada, Australia, Denmark, South Korea, Netherlands, Spain, United Kingdom
NCT04802590	Ibrutinib + CD20 Antibody + Venetoclax	II	MCL	Minimal Residual Disease (MRD) Rate	Recruiting	2026-03-31	Belgium, France, United Kingdom

Recent breakthroughs in universal CAR-T (UCAR-T) technology drive cell therapy toward an “off-the-shelf” model. This therapeutic strategy utilizes gene editing to ablate endogenous TCR and MHC molecules in T cells, generating universal cellular products compatible with multiple patients. Preliminary clinical trials have demonstrated both the efficacy and safety of this approach in treating lymphoma. Based on this foundation, research groups have engineered a modular universal chimeric antigen receptor T-cell (MU-CAR-T) platform incorporating an Sd/Gv covalent linkage system. This system enables the modular assembly of diverse single-chain variable fragments, facilitating flexible and precise multi-antigen targeting. MU-CAR-T cells have shown potent cytotoxic activity against HIV-infected and T-cell lymphoma cells, while also suppressing tumor progression *in vivo* through enhanced immune infiltration and cytokine release (80). This modular framework streamlines manufacturing processes and quality control protocols and establishes a comprehensive therapeutic paradigm with broad applicability to oncological and viral pathologies.

To optimize immunocompatibility, Zhu S and his team used lentiviral transduction technology. They developed CD38-targeted UCAR-T cells overexpressing *LLT1*. The introduction of *LLT1* promotes CAR-T cell proliferation and antitumor activity. It also effectively defends against rejection by allogeneic NK and T cells. This improved UCAR-T demonstrates higher survival rates and tumor clearance capacity. It reduces inflammatory responses, providing a key solution to the immune rejection problem in universal therapies (81). Deep mechanistic studies on host immune rejection have led to Dasatinib-resistant UCAR-T (KM UCAR-T) development. This involves introducing the *T316I* mutation in the *LCK* gene and knocking out the *TRAC* and *B2M* genes. When combined with a Dasatinib administration strategy, this approach has led to the development of a novel cell therapy with both anti-rejection and anti-tumor properties (82). *In vitro* studies have demonstrated that KM UCAR-T cells retain their activation and functional capacity in the presence of Dasatinib. Furthermore, in xenograft mouse models, these cells show effective tumor clearance and resistance to host immune responses (82). The integration of multiple strategies (multiplex antigen recognition, immune modulation, and optimization of manufacturing processes) has significantly expanded the clinical potential of UCAR-T therapies.

#### 3.2.2 Expansion of immune checkpoint inhibitors

Immune checkpoint therapy has introduced a transformative approach to cancer treatment by modulating T cell activation pathways and reshaping the anti-tumor immune response. Beyond the established targets of CTLA-4 and PD-1/PD-L1, identifying emerging immune checkpoints such as LAG-3, TIM-3, and TIGIT offers promising avenues to overcome current therapeutic limitations and improve clinical efficacy (83). LAG-3 acts as a key co-inhibitory receptor that inhibits T-cell function by binding to MHC-II molecules (84, 85). LAG-3 and PD-1 work synergistically to drive the exhaustion of CD8^+^ T cells, suppressing the IFN-γ-dependent anti-tumor immunity. Blocking both pathways simultaneously can promote IFN-γ release. It reduces T-cell exhaustion and enhances tumor clearance (86). Relatlimab is the first FDA-approved LAG-3 inhibitor. It is combined with a PD-1 antibody to improve efficacy (87). In R/R HL patients who failed PD-1 treatment, the combination of Relatlimab and Pembrolizumab achieved an ORR of 31%, and the PFS at 12 months reached 39%. Dual blockade of the LAG-3/PD-1 pathway can reverse the exhaustion of CD8^+^ T cells, promote the release of IFN-γ, and enhance the tumor clearance ability (88).

TIM-3 acts as a transmembrane immunomodulatory molecule. It drives T cell function exhaustion by binding ligands such as Galectin-9. Its inhibitor, sabatolimab, has shown significant potential in the treatment of myeloid tumors (89–91). The recently developed small-molecule compound ML-T7 effectively disrupts PtdSer/CEACAM1 signaling, thus enhancing the functional activity of CAR-T cells. Moreover, by targeting the FG-CC0 cleft of TIM-3, ML-T7 contributes to remodeling the immune microenvironment. Its therapeutic efficacy as a monotherapy is comparable to that of TIM-3-targeting antibodies, highlighting its potential as a promising immunomodulatory agent. The combination of PD-1 inhibitors can significantly improve the tumor suppression rate. It has the advantage of oral administration (92). It has been further found that the TIM-3/Galectin-9 signaling axis forms a negative feedback loop. This occurs through IFN-γ-mediated upregulation of Galectin-9. This finding provides theoretical support for developing combination immunotherapy (93).

TIGIT, a co-inhibitory receptor that interacts with CD155 and CD112, plays a critical role in immune evasion by suppressing the activity of NK cells and T lymphocytes. Its underlying mechanisms and therapeutic potential in lymphoma have increasingly attracted research interest, positioning it as an emerging focus in tumor immunology (94, 95).In FL, TIGIT is significantly overexpressed in tumor-infiltrating T cell subsets. These include Treg, follicular helper T cells, and exhausted T cells. It is closely related to a poor survival prognosis. Importantly, anti-PD-1 therapy can specifically regulate TIGIT^+^ depleted T cell subsets. This suggests the potential value of a dual-target blockade strategy (96). The mechanism of recurrence after CAR-T therapy in mantle cell lymphoma showed that TIGIT expression was significantly upregulated on depleted T cells and cytotoxic T cells (CTLs). Tumor cells use monocyte-expressed CD155/PVR to promote immunosuppressive signaling by upregulating *TIGIT* expression. This interaction is associated with the accumulation of MDSCs, collectively contributing to an immunosuppressive TME. These findings underscore the pivotal role of TIGIT in mediating resistance to CAR-T therapy (97). In DLBCL, the co-expression of TIGIT and PD-1 in tumor-infiltrating T cells (TILs) is particularly prominent. These TILs not only have close contact with malignant B cells but also reveal functional defects in cytokine secretion. Combined blockade of TIGIT and PD-1 can completely clear A20 lymphoma and significantly prolong survival in most mice. This highlights the clinical translation prospect of synergistic immune checkpoint inhibition (98). Further analysis of TME revealed that TIGIT was highly frequently expressed in TME cells from small B-cell lymphoma and ALCL, and its expression levels were significantly correlated with OS and PFS (99). In chronic lymphocytic leukemia/small lymphocytic lymphoma (CLL/SLL), negative expression of TME cells is associated with shorter OS. Subtype-specific differences exist in the co-expression patterns of *TIGIT* and *PD-L1*. These differences provide a molecular basis for accurately screening the beneficiary population for TIGIT inhibitor combined with PD-1/PD-L1 blockade (99).

These novel immune checkpoints and the classic PD - 1/CTLA - 4 form a multi-dimensional regulatory network. Their combined blockade strategies have demonstrated the potential to enhance the anti-tumor immune response in pre-clinical models. Current research centers on precisely identifying predictive biomarkers, developing novel combinatorial strategies, such as co-administration of TIM-3 inhibitors with PD-1 blockade, and exploring new drug modalities, including the small-molecule agent ML-T7. These efforts aim to address the clinical limitation of suboptimal response rates associated with existing immune checkpoint inhibitors and to establish a foundation for next-generation tumor immunotherapy paradigms.

### 3.3 Combination therapy strategies

The innovation of combination therapy strategies in lymphoma has significantly improved clinical outcomes. The synergistic integration of immune checkpoint inhibitors (ICIs) and antibody-drug conjugates (ADCs) has shown significant therapeutic benefits. ADCs enable targeted delivery of cytotoxic agents, facilitating precise tumor cell elimination and increased antigen exposure, while anti-PD-L1 antibodies alleviate tumor-induced immunosuppression. This combination significantly amplifies anti-tumor efficacy compared to monotherapies. However, the underlying mechanisms of immunogenic cell death and the potential toxicity risks associated with such regimens require further comprehensive investigation.

In epigenetic regulation, the histone deacetylase (HDAC) inhibitor Romidepsin is combined with PD-1 antibodies. This combination can upregulate chemokines to promote T-cell infiltration. It significantly activates CD4^+^/CD8^+^ TILs. The combination achieves synergistic tumor inhibition in B-cell lymphoma models (100). Ruan J et al. achieved an ORR of 65.2% in patients with previously untreated PTCL using a chemotherapy-free regimen of Romidepsin and Lenalidomide. In the AITL subtype, the ORR increased to 78.6%. The two-year PFS and OS outcomes were comparable to those of the standard CHOP chemotherapy regimen. These findings suggest a viable therapeutic alternative for elderly patients or those unsuitable for conventional chemotherapy (101). In a phase I/II study, the PD-1 antibody Sintilimab was combined with the histone deacetylase inhibitor Chidamide. This combination was used to treat relapsed/refractory NK/T-cell lymphoma. It resulted in an increased ORR of 59.5% and a CR rate of 48.6%. These results were significantly better than historical single-agent data (102).

Metabolic intervention strategies focus on reversing the immunosuppressive microenvironment. The IDO inhibitor Epacadostat restores T/NK cell functionality by disrupting the tryptophan–kynurenine metabolic axis, reducing plasma kynurenine levels by up to 90% (103–105). Moreover, *IL4I1*-mediated oxidative metabolism of tryptophan has been shown to induce the expression of immunosuppressive molecules, including *PD-L1*, by activating aryl hydrocarbon receptor (AHR) signaling. Silencing of *IL4I1* expression significantly enhances the synergistic antitumor efficacy of *PD-1* blockade in combination with CD19-directed CAR-T therapy (106, 107). Furthermore, in a study evaluating the combination of Rituximab and Lenalidomide in treatment-naïve FL, resistance was mediated by the transcription factor *PD-1* through modulation of the PD-L1/4-1BBL signaling axis. Dual targeting of PD-L1/4-1BB antibodies can reverse drug resistance and promote dendritic cell maturation. This provides a new direction for FL therapy (108). Chamorro-Jorganes et al. found that combining EZH2 and BRD4 inhibitors showed significant antiproliferative effects. They achieve this by synergistically blocking *MYC* transcription and inducing G1 phase arrest. YPEL2 was identified as a key factor influencing the efficacy of dual targeting (109).

Significant advances have been made in developing innovative treatment models through integrating physiotherapeutic approaches with immunotherapy. This combination has yielded promising breakthroughs, enhancing therapeutic outcomes. In a CAR-T bridging study for patients with R/R DLBCL, the one-year PFS (51.2%) and OS (86.7%) in the radiotherapy bridging group were significantly better than those in the chemotherapy bridging group (28.2%/52.7%). The treatment withdrawal and toxicity rates were also lower in the radiotherapy group. These findings suggest the unique value of local radiotherapy in optimizing the sequential regimen of CAR-T therapy (110). These multi-dimensional combination strategies, from immune activation to metabolic regulation to developing innovative therapies, have made substantial breakthroughs in individualized lymphoma treatment.

### 3.4 Emerging fields

#### 3.4.1 Microbiome - tumor axis

Growing insights into the TME have increased focus on the mechanistic interaction between microbial populations and tumor dynamics. The intestinal microbiota has emerged as a key modulator in the onset, progression, and therapeutic responsiveness of lymphoma. Its influence on tumor immune microenvironment homeostasis is exerted through several regulatory pathways, including modulation of host immune responses, metabolic output, and pro-inflammatory signaling networks (111).

Routy B et al. found that fecal microbial transplantation (FMT) could enhance immunotherapy sensitivity. It does so by remodeling the microbiota structure. FMT intervention increased the response rate of PD-1 inhibitors in tumor-bearing mice by 2.3 times. The mechanism involved an increase in intestinal microbiota-mediated infiltration of CD8^+^ T cells. It also involved downregulating immunosuppressive factors such as IL-10 (112). The abundance of specific probiotics, such as Bifidobacteria and *Akkermansia muciniphila*, is positively correlated with the sensitivity of immunotherapy. Microbial metabolites such as short-chain fatty acids affect the progression of lymphoma by regulating the inflammatory pathway (113–115). In a clinical study (NCT04567446) involving 33 patients with B-cell lymphoma, the objective response rate of the *Akkermansia muciniphila* (Akk bacteria) high-abundance group (;1%) reached 78% after 6 months of CAR-T therapy. This rate was significantly higher than that of the low-abundance group. The abundance of Akk bacteria was positively correlated with CD8^+^ T cell infiltration and IFN-γ release in tumors/bone marrow. In the mouse model, the tumor volume of the Akk bacteria-supplemented group was reduced by 58%, 72%, and 65% on days 10, 34, and 46, respectively. Survival was also significantly prolonged in this group. Clinical translational studies have further verified the clinical value of microbiota regulation. In a multicenter cohort analysis, the diversity of the gut microbiota in patients with allogeneic hematopoietic cell transplantation (allo-HCT) was significantly correlated with survival. The high microbial diversity group showed a 29% to 51% reduction in mortality risk. Microbiota disruption was characterized by a significant loss of diversity accompanied by single-genus dominance. This pattern was a predictive indicator for developing graft-versus-host disease (116). Mendelian randomization studies have revealed species-specific effects from the perspective of causal associations. *Faecalibacterium prausnitzii* reduces the risk of Hodgkin lymphoma. Coprococcus antagonizes follicular lymphoma. Ruminococcaceae UCG-002 increases the risk of DLBCL by upregulating the inflammatory factor MIG (117, 118). Moreover, prospective studies of patients with DLBCL have shown the following. Dynamic changes in the abundance of Enterobacteriaceae during R-CHOP treatment are significantly associated with the risk of bacterial infection. An initial Enterobacteriaceae abundance greater than 4.5% is an independent prognostic indicator of infection risk. This observation supports the clinical adoption of early intervention strategies to preempt infection onset (119). These findings reveal the molecular mechanism by which the gut microbiota regulates the immune system against malignant tumors, which provides an important scientific basis for formulating personalized lymphoma treatment plans by adjusting the microbial community.

#### 3.4.2 Nanotechnology

Recent advancements in nanotechnology are paving the way toward personalized therapeutic strategies for malignant tumors. Among these innovations, nucleic acid-based drug delivery systems, particularly mRNA vaccines, have emerged as a prominent area of investigation. A main challenge in this domain is overcoming intracellular delivery barriers associated with nucleic acid molecules while achieving targeted accumulation within the tumor immune microenvironment.

Kranz LM et al. developed charge-optimized RNA lipid complexes (RNA-LPX) for delivery system optimization. These complexes trigger an immune response to virus-like infections through ligand-independent DC-targeting mechanisms (120). Sasaki K’s team manipulated the size of mRNA lipid nanoparticles (LNPs) to 200–500 nm by microfluidic technology, and the optimal formulation A-11-LNP was screened to significantly enhance the RNA uptake and antigen presentation efficiency of spleen DCs (121). Mannose-modified lipid nanoparticles (STLNPs-Man) can increase mRNA delivery efficiency by a factor of 4. This is achieved through the DC mannose receptor-mediated endocytosis pathway. They also enhance the synergistic effect with immune checkpoint inhibitors by downregulating T cell CTLA-4 expression. This downregulation of *CTLA-4* expression improves the combined efficacy (122). Moreover, a research group has engineered biomimetic nanoparticles cloaked with dendritic cell membranes (DPNs). They can enhance lysosomal escape efficiency through membrane fusion mechanisms (123). A team innovatively integrated a nanoplatform of CRISPR/Cas9 gene editing and photothermal therapy. It induces immunogenic cell death through *PD-L1* knockout synergistically with sub-high temperature. This significantly promotes DC maturation and cytotoxic lymphocyte infiltration (124).

For the mechanism of drug resistance, a research team has developed an RGD-targeted peptide nanoplatform. This nanoplatform can specifically deliver *Bcl-2* antisense oligonucleotides to αvβ3 integrin-high expression tumor cells. It achieves efficient gene silencing and adapts to various nucleic acid drugs (125). The nucleolin-targeted nanodrug PA-HM@DOX/ICG was functionalized with the AS1411 aptamer. This aptamer-based modification enables precise targeting of DLBCL cells by exploiting the overexpression of nucleolin on their surface. The combination therapy using this nanodrug resulted in a tumor inhibition rate of 91.5%. It also caused a significant reduction in toxicity (126). Carvalho S.M. and others integrated ZnS quantum dot imaging and CD20/CD19 double antibody targeting. This integration was based on a chitosan-functionalized nanoplatform. It made the apoptosis rate of NHL cells reach 82.4%. Meanwhile, the damage to normal B cells was less than 15% (127). Technological advancements, including improved drug delivery efficiency, integration of combinatorial therapeutic modalities, and the realization of synchronous diagnostic and therapeutic functions, have significantly expanded the application potential of nanomedicine in treating malignant tumors of the lymphatic system.

#### 3.4.3 Artificial intelligence-driven strategies

The rapid development of AI-assisted diagnosis and treatment platforms has pushed lymphoma to a new stage. AI technology integrates multi-omics data, such as genomics and transcriptomics, with deep learning algorithms. AI technology has achieved systematic innovation from molecular typing to treatment decision-making. Considering the EcoTyper framework as an example, a high-resolution TME map was constructed for the first time. This map contains 13 cell types and 44 cell states. It was created by collaboratively analyzing the bulk and single-cell RNA-seq data of hundreds of DLBCL cases. This breakthrough overcomes the limitations of traditional classification. It also reveals the dynamic interaction network between malignant B cells and TME (128). In therapeutic development, the Auto-RapTAC platform has significantly reduced the screening cycle for PROTAC molecules to just eight days by employing a modular, automated design. Using this high-throughput system, six potent degraders targeting CDK2, CDK12, and BCL6 have been successfully developed, thus substantially expediting the discovery and optimization of heterobifunctional drugs (129). AI can improve the efficiency of PROTAC development in the following ways. It uses machine learning to analyze structure-effect data and build predictive models. It performs virtual screening of potentially efficient candidates. It generates and evaluates the optimal design of new structures. It mines and analyzes experimental data to reveal key influencing factors. It assists in optimizing the synthesis route and improving preparation efficiency. For example, AlphaFold uses deep residual convolutional neural networks to efficiently capture complex patterns in protein data for accurate structure prediction. This provides key information for PROTAC design (130).

A deep learning-based whole-slide analysis tool for H&E staining demonstrated an overall accuracy of 0.932 in differentiating between FL, DLBCL, and CHL. Its multi-class diagnostic performance was comparable to that of expert pathologists and offered enhanced interpretability through heat map visualization. Virtual H&E staining technology achieved a staining quality pass rate of 92%, with diagnostic concordance closely matching that of conventional chemical staining (90% vs. 92%). These advancements collectively lay a robust foundation for the transition toward digital pathology (131, 132). (https://xulymphoma.shinyapps.io/PCDI_pred/) (127).

In terms of prognosis prediction, researchers integrated multi-omics data from 339 DLBCL patients. They aimed to construct a PCD index model. This model dynamically correlates ctDNA burden, minimal residual disease (MRD) status, and immune microenvironment characteristics. It does so through an online tool. The tool is designed to accurately identify patients with chemotherapy resistance to R-CHOP. It also guides targeted therapy selection (https://xulymphoma.shinyapps.io/PCDI_pred/) (133). Ferrández M.C. et al. developed a deep learning PET/CT prognostic model. This model demonstrated an area under the characteristic curve (AUC) of 0.71 in 1132 patients. The performance was significantly better than the International Prognostic Index (IPI). The model can predict treatment response without tumor segmentation (134). Song C’s team built a multimodal model based on 2.5D transfer learning. The integration of clinical features with radiomics data has enabled the development of a predictive model capable of accurately differentiating lymphoma from tuberculous lymphadenitis in patients with HIV/AIDS, achieving an AUC of 0.920 (135). AI has driven transformative progress in multiple pivotal areas, including elucidating molecular pathogenesis, accelerating drug discovery pipelines, and optimizing clinical decision-making processes. These advancements are progressively shaping a comprehensive precision medicine paradigm for lymphoma, thus promoting individualized diagnostic and therapeutic interventions from early detection through post-treatment monitoring.

#### 3.4.4 Liquid biopsy: ctDNA mutation profiling to guide treatment decisions

ctDNA can be an important indicator for liquid biopsy. Analysis of the ctDNA mutational spectrum enables clinical target identification, dynamic monitoring of therapeutic efficacy, and early detection of disease recurrence. The ctDNA-based assessment of MRD has shown substantial advantages in individualized lymphoma treatment. Due to its high-sensitivity detection capabilities, this approach allows real-time tracking of tumor dynamics, thus providing timely and reliable data to inform and refine clinical decision-making.

In FL, high-sensitivity droplet digital PCR (ddPCR) revealed an EZH2 mutation frequency of 41.5%, significantly higher than the 27% detected through conventional single-point tissue biopsy. Liquid biopsy further identified undetected mutations in an additional 14 tissue samples. Dynamic monitoring showed that ctDNA levels in treatment-responsive patients declined by over 100-fold. Mutations could be detected and reproduced 6 months before recurrence, and EZH2 wild-type clones were more likely to infiltrate the bone marrow. This has expanded the scope of benefit for the precise use of inhibitors such as tazemetostat (136). In different lymphoma subtypes, ctDNA has distinct application values in disease monitoring and efficacy evaluation. For extranodal NK/T-cell lymphoma (ENKTL), ctDNA sequencing has identified *BCOR*, *TP53*, and *DDX3X* mutations associated with prognosis. The dynamics of these mutations can distinguish between remission and relapse (137). Intravascular large B-cell lymphoma (IVLBCL) is characterized by significantly elevated ctDNA concentrations compared to DLBCL. High-frequency mutations are frequently observed in MYD88 (56%) and CD79B (44%), with *BCL6* mutations significantly enriched in cases with CNS involvement (138). In PTCL, ctDNA was detected in 95.7%, one-year PFS and OS were significantly reduced in the high-concentration group, and dynamic monitoring was consistent with imaging evaluation in 81.9% (139). ctDNA also plays a key role in predicting treatment response. Chen et al. developed a detection system for 29 TP53 mutation probes in patients with CAR-T-treated NHL. They found that the median PFS in the high ctDNA group was only 1.4 months. This was significantly shorter than that in the low ctDNA group (140). In cHL and DLBCL, ctDNA plasma concentrations were significantly negatively correlated with tumor mutational burden. Continuous monitoring of ctDNA could track clonal evolutionary trajectories in these malignancies. However, the threshold for ctDNA detection in FL needed to be optimized to improve clinical applicability (141, 142). Despite the limitations of some studies, such as small sample sizes and a constrained scope of detectable genetic alterations, blood-based ctDNA analysis has successfully addressed several inherent drawbacks of conventional tissue biopsies. In DLBCL, ctDNA technology enables simultaneous guidance of therapeutic regimen selection, dynamic monitoring of treatment responses, and early detection of disease recurrence. These capabilities substantially enhance the precision and continuity of clinical management, spanning the entire course from initial diagnosis to post-therapeutic surveillance.

#### 3.4.5 Dynamic risk stratification: real-time monitoring system based on ctDNA-MRD

The ctDNA-based assessment of MRD has shown substantial advantages in personalizing lymphoma treatment. Due to its high sensitivity and high-specificity detection capabilities, this modality enables real-time surveillance of tumor dynamics, thus providing timely and reliable molecular data to inform precise clinical adjustments to therapeutic strategies. The ctDNA-MRD real-time monitoring system can evaluate the treatment effect and predict recurrence by detecting changes in ctDNA levels, and guide the adjustment of treatment strategies. For patients with CAR-T-treated R/R LBCL, the median OS of ctDNA-positive patients at day 28 was only 6.7 months. None of the ctDNA-negative patients experienced disease progression. The sensitivity and specificity of MRD detection reached 83%-100%. This systematically verified the early warning value of ctDNA in evaluating CAR-T efficacy. Compared with traditional PET/CT, ctDNA can identify individuals at high risk of recurrence in advance and enable timely adjustment of treatment strategies (143). Soscia R et al. analyzed 73 patients with DLBCL. The analysis was based on MRD monitoring using immunoglobulin (IG) gene rearrangements in ctDNA. It further demonstrated the prognostic stratification efficacy of this monitoring method. MRD-negative patients had significantly better PFS than positive patients at mid-treatment and treatment end (144). This non-invasive detection technology not only enables effective stratification of patient groups based on prognostic risk but also facilitates clinical trial design incorporating dynamic risk adjustment. It holds significant promise as a foundational tool for developing personalized diagnostic and therapeutic strategies in clinical practice.

ctDNA mutation profiling and ctDNA-MRD real-time monitoring systems are essential technologies within liquid biopsy. Both methods are non-invasive and enable dynamic disease assessment. ctDNA mutation profiling is primarily used for early detection and prediction of treatment response, while ctDNA-MRD monitoring is applied during therapy to track disease progression and provide early relapse warnings. Despite their application at distinct clinical stages, these methodologies jointly contribute to advancing personalized medicine.

#### 3.4.6 Radiomics: PET/CT texture analysis to predict CAR-T efficacy

Positron emission tomography/computed tomography (PET/CT) is an image-based biomarker development method that enables accurate assessment based on the Lugano criteria through fusion imaging of metabolic activity (PET) and anatomical structure (CT) (145). However, traditional PET/CT detects lesions based on macroscopic tumor burden. This approach may miss microscopic residual lesions and lead to a false negative risk. It also has limitations such as radiation exposure and high economic costs. With the development of radiomics technology, in-depth analysis based on texture features is breaking through the traditional evaluation framework. The radiomics model established by Kim JJ et al. showed improved predictive accuracy relative to traditional parameters, such as metabolic tumor volume (MTV), in evaluating the therapeutic response to axi-cel CAR-T therapy in patients with relapsed or refractory DLBCL. The high shape complexity (PC value) of non-round/irregular nodules is strongly associated with poor prognosis. This provides a new dimension for non-invasive evaluation of immunotherapy response (137). In another study of mediastinal lymphoma subtypes (GZL/PMBCL/cHL), FDG-PET texture analysis was performed. It revealed that the metabolic activity of primary mediastinal large B-cell lymphoma (PMBCL) was significantly higher than that of other subtypes. The random forest model showed a dichotomous AUC of 0.87 for this analysis. The heterogeneous characteristics of PMBCL identified through this analysis could assist in pathological typing and puncture localization (146). These innovative methods transform medical imaging information into quantifiable diagnostic indicators by analyzing the differential characteristics of tumor cell metabolism. Although these technologies require further large-scale clinical validation and fundamental research, their application in disease stratification, treatment response assessment, and the development of personalized therapeutic strategies is driving innovation in clinical management models for lymphoma.

### 3.5 Others

#### 3.5.1 Design of the lymphoma cohort in the NCI-MATCH trial

The National Cancer Institute’s (NCI) Molecular Analysis Therapy Selection (MATCH) trial uses an adaptive design model. It detects gene mutations, copy number variations, and fusion events in tumor tissues based on next-generation sequencing (NGS) technology. The trial combines gain-of-function/deletion mutations to accurately match lymphoma patients with specific molecular markers to the corresponding targeted therapy group (147). The establishment of a nationwide network of clinical laboratories has enabled the standardization of testing protocols across institutional samples, thus validating the feasibility of large-scale molecular subtyping in clinical translational settings (148).

The NCI-MATCH trial has demonstrated substantial clinical value in managing various solid tumors, including breast and colorectal cancers. Based on the molecular classification expertise developed through this platform, research efforts are now focused on establishing a gene-guided therapeutic framework for lymphoma. This approach aims to identify more individualized treatment strategies for patients with relapsed or refractory disease by analyzing tumor-specific molecular alterations. In the NCI-MATCH trial, Mansfield AS et al. reported cross-tumor efficacy in rare malignancies harboring ALK or ROS1 rearrangements. Among patients with ALK rearrangements, an objective response rate of 50% and a median PFS of 3.8 months were observed, outcomes that significantly surpassed those achieved with conventional chemotherapy regimens (149). Treatment with tazemetostat targeting EZH2/SMARCB1 mutant subsets in pediatric lymphoma resulted in disease control for six months or more. One patient experienced a sustained objective response. This demonstrates the therapeutic potential of epigenetic regulators in specific molecular subtypes (150). The trial uses a multi-arm design and integrates experts from different fields to simultaneously verify the effectiveness of other therapies. It accelerates the scientific research process and enhances the adaptability of treatment options, helping lymphoma patients obtain more personalized disease management strategies.

#### 3.5.2 Real-world evidence: application of the flatiron health database in efficacy validation

The incorporation of real-world evidence (RWE) into clinical research on lymphoma has emerged as a critical paradigm for validating therapeutic efficacy. The Flatiron Health database, an advanced platform integrating electronic health records (EHRs) with structured diagnostic and treatment data, offers distinct advantages. Its robust data mining capabilities enable comprehensive, multi-dimensional analyses, thus enhancing the evaluation of the clinical value and effectiveness of lymphoma treatment strategies. In a real-world cohort study involving 4336 patients with MCL, the median real-world overall survival (rwOS) was 35 months. This rwOS was significantly better than that observed with conventional chemotherapy. A high-risk subgroup (5% of the cohort) harboring a 17p deletion/TP53 mutation had a poor prognosis. BTK inhibitors have shown the capacity to partially reverse adverse outcomes in this subgroup, providing an evidence-based treatment option for older patients or those unable to tolerate intensive chemotherapy (151). In primary DLBCL, Breinholt et al. demonstrated that the prognostic relevance of *TP53* mutations is consistent across cohorts. However, the clinical significance of *PRDM1* and *NOTCH2* mutations remains to be validated in larger patient populations (152). These findings highlight the need for dynamic optimization of molecular classification systems based on real-world data and evolving biological insights. Similarly, studies of FL and histological transformation (HT) have revealed a significant increase in the heterogeneity of tumor mutational burden and driver genes (such as EZH2, and CREBBP) as the disease progresses. This increased heterogeneity may affect the use of targeted drugs (such as EZH2 inhibitors). It highlights the need for dynamic monitoring of molecular profiles before treatment (153). The research method based on actual diagnosis and treatment records can use massive case information to evaluate the efficacy and safety of drugs and combine genetic testing results with patient recovery data to promote the discovery of new disease surveillance indicators.

## 4 Challenges and future directions

Currently, the precision diagnosis and treatment system of lymphoma still faces multiple challenges. The molecular markers used to diagnose lymphoma are not comprehensive, and some rare subtypes and complex cases still have blind spots. The CD20 marker, commonly used in diagnosing and treating B-cell lymphomas, demonstrates reduced discriminatory efficacy in certain pathological subtypes, such as double-expression and triple-hit lymphomas. For instance, in approximately one-third of MCL patients receiving BTK inhibitors, acquired resistance contributes to disease progression. This resistance is often associated with mutations at the C481S locus of the BTK protein, which impair drug binding and therapeutic efficacy. It also includes VLA-4 integrin-mediated cell adhesion escape (154). These drugs can cause life-threatening side effects, such as severe cardiotoxicity (155). Moreover, the dynamic remodeling of the tumor microenvironment further exacerbates treatment resistance, such as inadequate CD8^+^ T cell infiltration, which is significantly associated with chemotherapy resistance to CHL (156). There are also bottlenecks in the field of immunotherapy, with objective response rates of only 60%-70% with ICIs in CHL and secondary resistance in approximately 40% of patients (157, 158). Despite the transformative efficacy of CAR-T cell therapy, its personalized manufacturing process is complex and economically demanding. Although approved and implemented in clinical settings across various developed countries, the widespread adoption of CAR-T therapy in developing regions remains limited due to financial and infrastructural barriers. In technological accessibility, NGS is an important tool for molecular typing; however, its routine clinical application is still restricted by challenges including sample integrity, detection sensitivity, and high associated costs. For example, traditional fluorescence *in situ* hybridization (FISH) technology requires high-quality samples. Flow cytometry is limited in identifying low-abundance abnormal cells. The demand for data storage and analysis of single-cell sequencing technology has increased exponentially (159–161). Moreover, clinical research is limited by data fragmentation. It is difficult to integrate cross-institutional case data and biological samples, especially for rare subtypes with an incidence rate of less than 1%. The insufficient sample size seriously limits the analysis of molecular characteristics and the optimization of treatment strategies. The advancement of precision diagnostics and therapeutics for lymphoma requires integrative, multi-dimensional innovation. A key strategy involves systematically exploring novel molecular biomarkers in parallel with an in-depth characterization of the metabolic phenotypes of lymphoma cells. This approach may enable the identification of distinct metabolic molecules, thus providing valuable insights and novel targets to improve diagnostic precision. For example, Göbel C et al. found that dual inhibition of the epigenetic regulators DOT1L and EZH2 reversed the MYC-driven germinal center B cell malignant phenotype, providing a new differentiation therapeutic pathway for DLBCL (GCB subtype) (162). Garcia-Lacarte M et al. found that the dual mechanism of action of the IL-10 signaling pathway in the microenvironment (maintaining malignant B cell survival and inducing T cell exhaustion) revealed a novel predictor of PD-1 inhibitor resistance (163).

Besides, optimizing detection methodologies and advancing more sensitive and specific genetic analysis technologies, such as digital polymerase chain reaction (digital PCR), can improve the accuracy of low-frequency mutation identification by enabling absolute quantitative detection (164). Improve flow cytometry and other diagnostic techniques, such as improving the ability to identify abnormal cell populations by optimizing antibody combinations and detection parameters. However, AI-based DNA methylation marker panels (MDMs) can identify over 80% of NHL cases, including early-stage lesions, while maintaining 90% specificity (165). Simultaneously, new therapies, such as PROTAC technology, can degrade the BTK protein and circumvent the impact of the C481S mutation. It has been found that *DNMT3A* promotes oxidative phosphorylation (OXPHOS) by activating the MEF2B/MYC axis, driving Ibrutinib resistance. Low-dose Decitabine targeting DNMT3A can restore drug sensitivity and provide a new strategy for reversing drug resistance (166). Construct a microenvironment regulation network to reverse the polarization state of TAMs. Targeting key glycolytic enzymes (e.g., LDHA, ENO2) offers a strategy to remodel the metabolic microenvironment of lymphoma. Pharmacological interventions aimed at stromal and immune cells within the tumor microenvironment may attenuate their protective influence on malignant cells. Similarly, the identification of novel immunotherapeutic targets and strategies is essential. Advancing the development of dual- or multi-target CAR-T and UCAR-T therapies holds promise for improving efficacy while reducing manufacturing costs. Furthermore, efforts to develop personalized lymphoma vaccines represent a pivotal direction for achieving individualized and cost-effective therapeutic solutions. China has established a leukemia diagnosis and treatment registry system. China has also established the Chinese Lymphoma Standard Dataset (2021 Edition). The Japanese Society of Hematology has published the Guidelines for Data Collection of Hematological Tumors. These efforts have laid the foundation for building a cross-institutional lymphoma diagnosis and treatment data platform (167). The establishment of a multi-institutional database, underpinned by the standardization of clinical diagnostic data, therapeutic interventions, and molecular testing as per the internationally recognized protocols, constitutes a foundational element in advancing precision medicine. This structured integration of diverse diagnostic, therapeutic, and multi-omics datasets enables coherent data aggregation across institutions, facilitating large-scale translational research and informing individualized clinical decision-making with improved accuracy and consistency. Next, a dynamic data quality control mechanism will be set up. Programs will be written to regularly check the completeness of the data, and statistical methods will be used to ensure consistency between data from different medical institutions. The establishment of a polycentric governance framework is essential to ensure regulatory compliance, operational efficiency, and clearly defined data ownership and collaboration protocols across institutions. Concurrently, developing advanced analytical tools is necessary to convert unstructured data, such as clinical text and imaging, into structured, analyzable formats. These tools will contribute to an open-source, reusable lymphoma data standardization toolkit, promoting broader applicability. A multi-center database, modeled after The European LeukemiaNet, should integrate pathological, molecular, genetic, therapeutic, and long-term follow-up data. Such a platform would enable high-resolution data analysis to support treatment response prediction and the identification of novel lymphoma subtypes. Under strict adherence to patient privacy protections, international research collaborations between academic institutions and industry may be formalized through cooperative agreements. These partnerships will facilitate advancing precision medicine initiatives for lymphoma, promote global academic exchange, and accelerate the collective development of personalized therapeutic strategies worldwide.

## Glossary

HL: Hodgkin Lymphoma
NHL: Non-Hodgkin Lymphoma
DLBCL: Diffuse Large B-Cell Lymphoma
FL: Follicular Lymphoma
MCL: Mantle Cell Lymphoma
PTCL: Peripheral T-Cell Lymphoma
ALCL: Anaplastic Large Cell Lymphoma
AITL: angioimmunoblastic T-cell lymphoma
COO: vell of origin
GCB: germinal center b-cell
DHL: double-hit lymphoma
CNV: copy number variations
CDKN2A: cyclin-dependent kinase inhibitor 2A
PFS: progression-free survival
OS: overall survival
DNMTs: DNA methyltransferases
HSCs: hematopoietic stem cells
PRC2: polycomb repressive complex 2
H3K27me3: trimethylation of lysine 27 on histone H3
PVT1: plasmacytoma variant translocation 1
TME: tumor microenvironment
TAMs: tumor-associated macrophages
IL-10: interleukin-10
TGF-β: transforming growth factor-beta
ENO2: enolase 2
cHL: classical Hodgkin lymphoma
scATAC-seq: single-cell assay for transposase-accessible chromatin using sequencing
BTK: bruton’s tyrosine kinase
EZH2: zeste homolog 2
EMA: european medicines agency
Cys: cysteine
ORR: objective response rate
CNS: central nervous system
BCR: B-cell receptor
NF-κB: nuclear factor kappa-light-chain-enhancer of activated B cells
SAM: S-adenosylmethionine
PROTAC: protein degrader targeting chimera
CTCL: cutaneous T-cell lymphoma
FDA: food and drug administration
ADCC: antibody-dependent cellular cytotoxicity
ADCP: antibody-dependent cellular phagocytosis
MTD: maximum tolerated dose
AE: adverse event
DLT: dose-limiting toxicity
CMR: complete metabolic response
MRD: minimal residual disease
UCAR-T: universal CAR-T
CTLs: cytotoxic T lymphocytes
MDSCs: myeloid-derived suppressor cells
TILs: tumor-infiltrating lymphocytes
CLL: chronic lymphocytic leukemia
SLL: small lymphocytic lymphoma
ICIs: immune checkpoint inhibitors
ADCs: antibody-drug conjugates
CR: complete response
AHR: aryl hydrocarbon receptor
FMT: fecal microbiota transplantation
allo-HCT: allogeneic hematopoietic cell transplantation
LAG-3: lymphocyte activation gene-3
TIM-3: T-cell immunoglobulin and mucin-domain containing-3
TIGIT: T-cell Immunoreceptor with ig and ITIM domains
IFN-γ: interferon-gamma
AI: artificial intelligence
AUC: area under the curve
IPI: international prognostic index
ctDNA: circulating tumor DNA
ENKTL: extranodal NK/T-cell lymphoma
IVLBCL: Intravascular large B-cell lymphoma
PET/CT: positron emission tomography/computed tomography
NCI-MATCH: national cancer institute molecular analysis for therapy choice
NGS: next-generation sequencing
RWE: real-world evidence
HER: electronic health records
